# The Path from Childhood Emotional Maltreatment to Disordered Eating Behaviors: The Role of Reflective Functioning and Food Addiction

**DOI:** 10.3390/nu17111863

**Published:** 2025-05-29

**Authors:** Alessandro Alberto Rossi, Andrea Tagliagambe, Anna Scuderi, Laura Dalla Ragione, Stefania Mannarini

**Affiliations:** 1 Department of Philosophy, Sociology, Education, and Applied Psychology, Section of Applied Psychology, University of Padua, 35131 Padua, Italy; 2 Center for Intervention and Research on Family Studies—CIRF, Department of Philosophy, Sociology, Education, and Applied Psychology, Section of Applied Psychology, University of Padua, 35131 Padua, Italy; 3 Residence Cabrini DCA, 54027 Pontremoli, Italy; 4 Eating Disorders Services-USL N1 “Palazzo Francisci”, 06059 Todi, Italy; 5 Food Science and Human Nutrition Unit, University Campus Biomedico of Rome, 00128 Rome, Italy

**Keywords:** food addiction, childhood traumatic experiences, emotional maltreatment, binge eating, uncontrolled eating, reflective functioning, low diet control, eating addiction

## Abstract

**Introduction**: Childhood emotional maltreatment, which includes emotional abuse and neglect, has been identified as a significant risk factor for the development of disordered eating behaviors related to overeating and reduced dietary control. At the same time, the literature suggests that childhood emotional maltreatment appears to be linked to deficits in reflective functioning which, in turn, may increase vulnerability to dysregulated, impulsive, and addictive behaviors. However, to date, the role of a key factor, such as food addiction (FA), within this model has not yet been investigated. Therefore, the aim of this study was to test a mediation model in which emotional abuse and neglect predict overeating and reduced dietary control through deficits in reflective functioning and FA symptoms. **Method**: Using a cross-sectional design, a conventional non-clinical sample of 543 participants was recruited and completed a set of standardized questionnaires. A multiple mediation model with observed variables was specified (10,000 bootstrap resampling). **Results**: The multiple mediation model showed good results, confirming the initial hypotheses. Specifically, emotional abuse and neglect were associated with FA symptoms through deficits in reflective functioning. In turn, FA symptoms predicted overeating behaviors and poor dietary control. **Discussion**: These findings highlight the central role of FA in linking reflective functioning deficits to disordered eating patterns associated with excessive food consumption. This study advances our understanding of the psychological mechanisms underlying disordered eating behaviors and underscores the need for targeted interventions addressing reflective functioning deficits and food addiction in individuals with a history of childhood emotional maltreatment.

## 1. Introduction

The prevalence of disordered eating behaviors related to overeating and excessive food intake is constantly increasing. These dysfunctional eating behaviors are gaining growing clinical attention due to their strong association with overweight and obesity [1,2,3,4], two complex chronic conditions arising from a multifaceted relationship of medical, environmental, and psychological factors [5,6]. These disordered eating behaviors are often linked to the overconsumption of highly processed and palatable foods. Indeed, highly processed foods (HPFs; especially those high in salt, sugar, and fat [7,8,9]) [7,8,10] are often associated with this growing global overweight and obesity epidemic [11,12], and they play a key role in the development and maintenance of obesity due to their addictive properties [13,14].

The continued and repeated consumption of these HPFs could contribute to the development and the maintenance of a dependency response in some individuals, namely, food addiction (FA) [10,15,16,17,18], that frequently lead to disordered eating behaviors related to excessive food intake [19]. Indeed, the potentially addictive nature of these palatable HPFs activates specific brain circuitry [20] and neural-reward pathways [21,22,23]. Consequently, in addition to sharing features typical of substance-related and addictive disorders (SRADs), FA also encompasses transdiagnostic aspects characteristic of eating disorders (EDs) [24,25], which mutually reinforce each other in a vicious cycle.

Indeed, individuals with (symptoms of) FA often experience tolerance that requires consuming larger amounts of food to achieve the same level of satisfaction [26] to seek the same sensations of pleasure and well-being [24,27]. Moreover, in individuals with FA, withdrawal-like symptoms may arise when the consumption of HPFs remains unfulfilled [28,29], leading to craving thoughts and behaviors, which, in turn, could lead to uncontrolled eating [19]. A defining feature of FA is its association with specific eating thoughts and behaviors [19], characterized by a lack of control over food intake and dietary patterns, with a wide range of eating behaviors, not limited to binge eating alone [27,30,31], that often hinder dietary control and healthy eating habits [27,31,32,33]. Indeed, FA is largely associated with behavioral manifestations related to food intake, ranging from grazing (i.e., repetitive and compulsive consumption of small amounts of HPFs) [27] to habitual overeating, culminating in binge-eating episodes and uncontrolled food consumption [19,34,35].

Moreover, FA symptoms can manifest across various ED-related conditions and diagnoses [24,25], further highlighting both its dual nature [36,37,38,39,40,41] and its complexity: on the one hand, medical and psychological characteristics related to SRADs [20], and on the other hand, psychological processes and behaviors related to EDs [19].

However, although the scientific literature suggests a complex interplay between genetic, medical, environmental, and psychological factors [1,42], little is still known about its etiology, its triggering features, and the psychological facets and factors that may contribute to its onset. Consequently, considering the dual nature of FA, it can be hypothesized that FA may share the etiological factors of both its components (SRADs and EDs). In this regard, a common factor linking EDs and SRADs appears to be childhood traumatic experiences (CTEs) [43,44,45,46,47,48,49,50].

In this regard, although studies are still limited, the scientific literature suggests a link between CTEs and FA [42,51,52,53,54], suggesting that the severity of the traumatic experience is associated with a more severe intensity of FA symptoms [55]. However, the literature does not seem to show consistent results in this regard, suggesting that not all types of CTEs appear to carry the same level of risk for the onset of FA [42]. Indeed, the literature appears to be inconsistent regarding the role and impact of sexual and physical abuse in contributing to the onset of FA [56,57]. However, the same studies seem to agree on the active role of emotional maltreatments—central facets of CTEs with deleterious effects on adult mental health [58]—highlighting their function as non-modifiable predictors and their influence as distal risk factors for the onset of FA [42,59].

Childhood emotional maltreatment refers to a recurring pattern of dysfunctional caregiver behavior [60], which could be characterized by emotional abuse and/or emotional neglect. On the one hand, childhood emotional abuse involves intentional acts by parents that humiliate and degrade the child, reflecting a failure of care through harmful actions. On the other hand, childhood emotional neglect arises from parental inaction, where essential needs such as support, attention, affection, and care are not adequately met [61,62,63,64]. Research suggests that both emotional abuse and/or emotional neglect appear to be directly and indirectly associated with the onset and maintenance of disordered eating behaviors [60,65], as well as for FA [56,57].

Additionally, childhood emotional maltreatment appears to be related to various consequences affecting individual development, including deficits in reflective functioning [44,66,67]. According to Fonagy, reflective functioning refers to an individual’s ability to mentally represent and understand human behavior by attributing it to underlying mental states, such as intentions, desires, beliefs, and emotions [66,67]. This capacity develops through the early caregiver–child relationship [67] and aligns with the broader concept of mentalization, which encompasses the imaginative process of interpreting one’s own and others’ actions [68]. Moreover, several studies suggest that deficits in reflective functioning have been associated, on one hand, with childhood emotional maltreatment [44,69], and, on the other hand, with disordered eating behaviors [60,69], behavioral addictions [70], and SRADs [71].

Indeed, individuals with deficits in reflective functioning tend to experience reality in a different, prementalized manner [44,72]. In particular, those who have faced childhood emotional maltreatment and the consequent deficits in reflective functioning may struggle to differentiate between external reality and their internal mental states during stressful situations [71], as they perceive these states as disorganizing [61,67]. The literature suggests that individuals with deficits in reflective functioning, when arousal or stress levels increase, may rely on more unconscious, instinctive, and automatic processes, which require an immediate (often unmet) response [73,74], that may lead to greater emotional and behavioral dysregulation [75,76]. In turn, this leads the individual to cope with distressing and psychologically painful experiences by using their bodies [60] and/or an external object for self-regulation [77], such as through substance use and/or disordered eating behaviors.

In summary, it is possible that individuals who have experienced childhood emotional maltreatment may develop deficits in reflective functioning [60,61,67,69], which, in turn, seems to lead individuals to experience more impulsive and dysregulated emotions and behaviors [69,72,76]. These individuals, lacking the internal resources to self-regulate, may appear to require an external regulator, such as substance use and/or food intake [60,77,78]. Therefore, FA would play a central role, acting as a bridge and/or reinforcement between deficits in reflective functioning and the use of external regulators, promoting the engagement in dysfunctional behaviors like excessive food consumption and/or reduced dietary control.

However, only a few studies seem to have applied this model in its entirety. Moreover, even fewer studies appear to have applied it to the field of dysfunctional eating behaviors, such as overeating and reduced dietary control. Lastly, to the best of our knowledge, the role of FA in this model has never been tested before.

Consequently, based on the above-mentioned scientific literature, the aim of this study is to test a multiple mediation model in which childhood emotional maltreatments, i.e., emotional abuse (X1) and emotional neglect (X2), are associated with deficits in reflective functioning (M1), and this, in turn, is associated with FA symptoms (M2). Finally, FA symptoms are simultaneously associated with overeating behaviors (Y1) and reduced dietary control (Y2). Explicit hypotheses about each path (relationship) between variables were formulated:

**H1.**  *emotional abuse, emotional neglect, deficits in reflective functioning, symptoms of FA, overeating behaviors, and reduced dietary control are positively correlated with each other;*

**H2.**  *emotional abuse and emotional neglect predict FA via deficits in reflective functioning;*

**H3.**  *emotional abuse and emotional neglect predict overeating behaviors, and reduced dietary control via deficits in reflective functioning and symptoms of FA;*

In other words, it was hypothesized that the positive associations between emotional abuse and emotional neglect would be mediated by the presence of deficits in reflective functioning and symptoms of FA.

## 2. Methods and Materials

### 2.1. Procedure

The snowball sampling method [79] was employed to enroll a conventional non-clinical sample through social media channels [80]. The eligibility criteria were as follows: Participants had to be (A) at least 18 years old, (B) native Italian speakers, (C) need to provide complete responses, and (D) must complete an online informed consent form. The research obtained authorization from the Ethics Committee of the University of Padua (protocol no. 547a).

### 2.2. Sample Size Determination

The sample size was planned a priori according to the primary statistical analysis (i.e., path analysis). The “*n:q* criterion” was employed, which refers to the ratio of participants (*n*) to model parameters (*q*) [81]. A minimum of 10 participants *per* parameter was needed to achieve sufficient statistical power for the proposed model. Consequently, since the model included 21 parameters, at least 210 participants were enrolled.

### 2.3. Participants

A sample of 543 participants was recruited. The sample comprised 116 males (21.4%) and 427 females (78.6%), aged from 18 to 84 years (*mean* = 38.39, *SD* = 14.80) and a BMI ranging from 15.37 to 50.81 (*mean* = 24.486; *SD* = 5.38). Further details are reported in Table 1.

### 2.4. Measures

A demographic information form was used to collect age, biological sex, education level, marital status, and employment status. As reported in Table 1, participants were asked to indicate the presence of a diagnosis of ED, as well as to provide their weight and height to calculate their body mass index (BMI) [82].

#### 2.4.1. Traumatic Experiences Checklist (TEC)

The TEC [83] was used to evaluate the presence of traumatic experiences. The TEC consists of 29 items, each answered on a yes/no scale, where participants indicate whether they have encountered each type of traumatic event in their lifetime. The TEC investigates the occurrence of four primary traumatic categories: (A) emotional abuse and neglect, (B) threats to physical integrity, including physical abuse, life-threatening situations, pain, and severe punishment, (C) sexual harassment and abuse, and (D) other potentially distressing events [83,84,85,86,87]. Higher scores indicate greater exposure to traumatic experiences. The Italian version of the TEC [88] was used in this study. Moreover, in line with previous research [60] and considering the aim of the present study, only the emotional abuse (Emo. Abuse) and emotional neglect (Emo. Neglect) scales were used and provided adequate internal consistency with a McDonald’s omega for categorical data of 0.6 and 0.5 for the Emo. Abuse scale and the Emo. Neglect scale, respectively.

#### 2.4.2. Reflective Functioning Questionnaire 8 (RFQ-8)

The RFQ-8 [89] was administered to assess individuals’ reflective functioning, namely, the ability to understand their own and others’ mental states. The questionnaire consists of eight items rated on a seven-point Likert scale, ranging from “completely disagree” (1) to “completely agree” (7), requiring participants to indicate their level of agreement with statements related to mentalizing abilities. This questionnaire evaluates two opposing dimensions: certainty and uncertainty regarding mental states. For this study, the uncertainty scale of RFQ-8 was used to assess deficits in reflective functioning. Higher scores correspond to higher deficits in reflective functioning. The Italian adaptation of the RFQ-8 [90] was employed, demonstrating good internal consistency: McDonald’s omega = 0.8.

#### 2.4.3. The Yale Food Addiction Scale 2.0 (YFAS 2.0)

The YFAS 2.0 [15,91] is a self-report questionnaire designed to evaluate the frequency of food addiction symptoms over the past year. It consists of 35 items rated on an eight-point Likert scale, reflecting DSM-5 criteria for substance-use disorders (SUD) [26], including tolerance, withdrawal, craving, and emotional distress. Food addiction (FA) is determined using two scoring approaches: the symptom count score, which quantifies the number of criteria met, and the diagnostic score, which categorizes severity as No FA, mild FA, moderate FA, or severe FA based on impairment or distress. In this study, the symptom count score was used, where higher values indicate a greater number of endorsed symptoms. The Italian adaptation of the YFAS 2.0 [15,92] was employed, demonstrating good internal consistency, with a McDonald’s omega for categorical data of 0.9.

#### 2.4.4. The Addiction-like Eating Behavioral Scale (AEBS)

The AEBS [33] is a 15-item self-report measure designed to evaluate behavioral addiction related to eating. Responses are provided on a five-point Likert scale, ranging from 1 (strongly disagree/never) to 5 (strongly agree/always). The AEBS-IT comprises two subscales: “appetite drive” (AD), which captures heightened sensitivity to reward-related food cues and overeating behaviors, and “low dietary control” (LDC), which reflects difficulties in exerting inhibitory control over eating behaviors. A higher score indicates greater impairment in the respective dimension. The AEBS has demonstrated validity and reliability in assessing excessive food intake behaviors. This study utilized the Italian version of the AEBS [93], which exhibited good internal consistency, with McDonald’s omega of 0.9 and 0.9 for the AD and LDC subscales, respectively.

### 2.5. Statistical Analysis

Statistical analyses were performed using the R software environment (v 4.3.2), employing the lavaan, psych, and tidyverse packages. Consistent with prior research, preliminary analyses were conducted before testing the proposed multiple mediation model [81,94,95]. Additionally, correlation analyses (*r*) were conducted to assess the strength of associations between variables [94,96] and to identify excessively high correlations (*r* > |0.80|), which could indicate multicollinearity or redundant constructs [96,97,98]. Correlation coefficients were interpreted based on Cohen’s guidelines [99]. Furthermore, since multiple mediation models function as linear models, their underlying assumptions were examined, with no violations detected.

**Figure 1:**
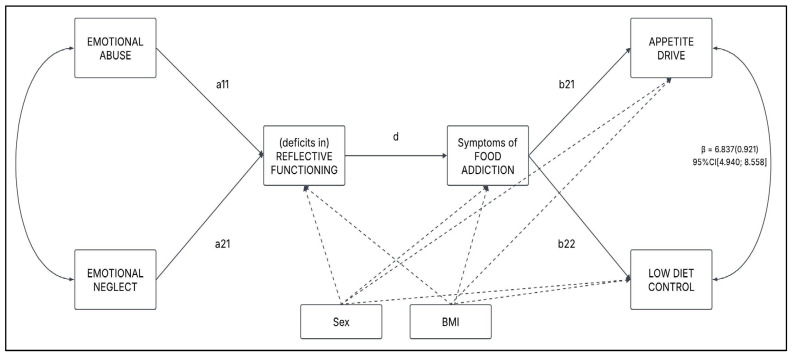
Path model—conceptual representation. Sex and BMI were included as covariates. *Note*: For the sake of clarity in presentation of this graph, indirect effects (e.g., emotional abuse → symptoms of food addiction) have not been included; only direct effects were depicted.

A multiple mediation model analysis with observed variables was performed [81,94,100]. More in detail, emotional neglect (X1) and emotional abuse (X2) were simultaneously regressed on problematic appetite drive (Y1) and low diet control (Y2) through the (deficits in) reflective functioning (M1) and FA symptoms (M2)—see Figure 1. Furthermore, according to the literature, sex and BMI were included in the model as covariates.

The maximum likelihood (ML) estimator was used. According to guidelines (e.g., Kline, 2023 [81]), a 10,000 non-parametric bootstrap resampling procedure was applied with the Bollen–Stine method for estimating confidence intervals [95,101,102,103], managing the non-perfect normality of some variables. The model was evaluated using goodness-of-fit indices and their recommended cut-off values from the literature [81,104]: χ^2^ (*p* > 0.050, *ns*); RMSEA (<0.080), CFI (>0.950), and SRMR (<0.080). However, it is important to note that several studies have shown that the RMSEA performs poorly when the number of degrees of freedom is very low (as in this case) [81,104,105,106], often resulting in excessively inflated and biased estimates (i.e., overly high values). All regression coefficients (β) reported in Section 3 were unstandardized.

## 3. Results

### 3.1. Preliminary Analysis—Correlations

The correlation analyses indicated that the psychological variables within the sequential multiple mediation model were positively interrelated (H1). However, none of the observed values exceeded the recommended threshold of |0.80|, allowing the statistical analyses to proceed. As shown in Table 2, correlation analyses revealed significant positive associations between all main study variables. Specifically, emotional abuse was positively correlated with emotional neglect *(r* = 0.408), reflective functioning deficits (*r* = 0.323), FA symptoms (YFAS2; *r* = 0.491), appetite drive (*r* = 0.317), and low diet control (*r* = 0.188). Emotional neglect was positively associated with reflective functioning deficits (r = 0.222), FA symptoms (*r* = 0.224), and appetite drive (*r* = 0.160), but only weakly with low diet control (*r* = 0.088). Reflective functioning deficits were positively correlated with FA symptoms (*r* = 0.321), appetite drive (*r* = 0.351), and low diet control (*r* = 0.236). FA symptoms were strongly associated with both appetite drive (*r* = 0.593) and low diet control (*r* = 0.309). Lastly, appetite drive and low diet control were moderately and positively correlated (*r* = 0.467).

### 3.2. Multiple Mediation Analysis

**Figure 2:**
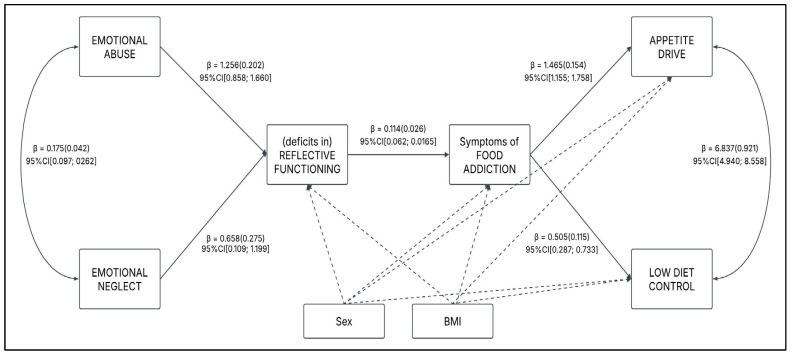
Path model with unstandardized regression coefficients, standard errors, and 95% confidence intervals. Sex and BMI were included as covariates. *Note:* For the sake of clarity in presentation of this graph, covariates and indirect effects (e.g., emotional abuse → symptoms of food addiction) have not been included; only direct effects were depicted.

The multiple mediation analysis (Figure 1 and Figure 2) supported the hypothesized results (see Table 3). The model showed a good fit to the data: χ^2^ (4) = 38.482, *p* < 0.001; RMSEA = 0.126 90%CI [0.092; 0.164]; CFI 0 0.958; SRMR = 0.057. Although the RMSEA exceeds the recommended threshold, it is worth noting that when the number of degrees of freedom is low, this fit index tends to perform poorly, often yielding excessively high values even in the presence of a well-fitting model. For these reasons, the model can be considered acceptable.

Emotional abuse (X1) was statistically associated with emotional neglect (X2): β = 0.175 (0.041), *p* < 0.001, 95%CI [0.099; 0.258]. Moreover, both emotional abuse (X1) and emotional neglect (X2) were positively associated with (deficits in) reflective functioning (M1). In particular, the strongest relationships were observed between emotional abuse (X1) and (deficits in) reflective functioning (M1), *path a11*: β = 1.256 (0.202), *p* < 0.001, 95%CI [0.858; 1.660], while a weakest relationships was observed between emotional neglect (X2) and (deficits in) reflective functioning (M1), *path a21*: β = 0.658 (0.275), *p* = 0.017, 95%CI [0.109; 1.199].

In line with hypotheses (H2), (deficits in) reflective functioning (M1) positively predict symptoms of food addiction (M2); *path d*: β = 0.114 (0.026), *p* < 0.001, 95%CI [0.062; 0.165].

Additionally, symptoms of food addiction (M2) were associated with both appetite drive (Y1) and low diet control (Y2). In particular, the strongest relationships were observed between symptoms of food addiction (M2) and appetite drive (Y1), *path b21*: β = 1.465 (0.154), *p* < 0.001, 95%CI [1.155; 1.758], while the weakest relationship was symptoms of food addiction (M2) and low diet control (Y2), *path a21*: β = 0.505 (0.115), *p* < 0.001, 95%CI [0.287; 0.733].

Lastly, a statistically significant positive association was found between appetite drive (Y1) and low diet control (Y2): β = 6.837 (0.921), *p* < 0.001, 95%CI [4.940; 8.558].

It is important to note that the covariate sex was not statistically associated with any of the main variables in the model. In contrast, the covariate BMI was significantly associated with FA symptoms, appetite drive, and low diet control, whereas it was not associated with reflective functioning (see Table 3).

Furthermore, an examination of the four total indirect paths was performed (H3). The first total indirect effect (controlling for X2 and Y2, X1 → M1 → M2 → Y1) was statistically significant: β = 0.210 (0.062), *p* = 0.001, 95%CI [0.102; 0.341]. The second total indirect effect (controlling for X2 and Y1, X → M1 → M2 → Y2) was statistically significant: β = 0.072 (0.027), *p* = 0.007, 95%CI [0.029; 0.133]. The third total indirect effect (controlling for X1 and Y2, X2 → M1 → M2 → Y1) was statistically significant: β = 0.110 (0.055), *p* = 0.048, 95%CI [0.015; 0.232]. The fourth total indirect effect (controlling for X1 and Y1, X2 → M1 → M2 → Y2) was not statistically significant: β = 0.038 (0.021), *p* = 0.065, 95%CI [0.005; 0.085].

The overall explained variance for the complete model was found to be 41.1% (*R*^2^ = 0.411) for appetite drive and 12.8% (*R*^2^ = 0.128) for low diet control. Detailed results are illustrated in Figure 2 and Table 3.

## 4. Discussion

In recent years, a growing number of studies have highlighted the significant impact of FA on both physical and mental health, considering its potential key role in the onset and maintenance of overweight and obesity through maladaptive eating behaviors such as grazing, emotional eating, and uncontrolled eating [1,19,27,107]. At the same time, numerous studies have focused on the crucial role of psychological variables, such as childhood emotional maltreatment and the resulting deficits in reflective functioning, that may underlie the development of disordered eating behaviors [60] related to overeating and reduced dietary control. However, to date, no model has yet been tested that integrates all these variables.

Therefore, the present study aimed to bridge this gap in the literature by testing a multiple mediation model, exploring how childhood emotional maltreatment (emotional abuse and neglect) influences FA symptoms through deficits in reflective functioning, ultimately contributing to overeating behaviors and reduced dietary control. Gaining insight into these mechanisms may shed light on the psychological factors underlying FA and contribute to the development of more effective interventions for those facing obesity and disordered eating behaviors related to excessive food consumption. This, in turn, could inform the design of timely psychological interventions and explore their effects on other constructs within research contexts.

The main findings of this study indicate that childhood emotional maltreatment, whether emotional abuse or emotional neglect, is not directly linked to overeating behaviors or reduced dietary control. Instead, the results highlight the central mediating role of deficits in reflective functioning and FA symptoms. Specifically, the relationship between emotional neglect and overeating behaviors is fully mediated by deficits in reflective functioning and FA. Interestingly, this pathway appears to account for a significant portion of the variance in overeating behaviors (*R*^2^ = 0.411). Similarly, the relationship between emotional abuse and reduced dietary control is also fully mediated by deficits in reflective functioning and FA. However, unlike the previous pathway, this one seems to explain a smaller proportion of the variance in dietary control behaviors (*R*^2^ = 0.128), suggesting that other factors may play a more substantial role in accounting for this phenomenon.

It is worth emphasizing that the model shows a good fit to the data. In fact, although the RMSEA exceeds the conventional threshold, it is important to note that this index tends to perform less reliably when the model has few degrees of freedom, often yielding inflated values despite otherwise adequate model fit [81,104,105,106], a conclusion further supported by other fit indices such as the CFI and SRMR. Due to this limitation, the overall fit of the model can still be considered satisfactory.

Although this is the first study to examine the combined behavior of these variables, these findings are consistent with the scientific literature and widely accepted theoretical frameworks, linking childhood emotive traumatic experiences, reflective functioning, SRAD, and EDs.

Individuals who have experienced childhood emotional maltreatment face an increased risk of developing psychiatric disorders as SRADs and EDs [46,54,108,109], via a deficit in reflective functioning. Indeed, these results suggest a positive association between childhood emotional maltreatment, both emotional abuse and emotional neglect, and deficits in reflective functioning, with the latter having a greater impact than the former. Indeed, several previous empirical studies have demonstrated that experiencing childhood emotional maltreatment, specifically in the form of emotional abuse and emotional neglect, is a key component of adverse and traumatic childhood experiences [60]. This type of maltreatment, persistently characterized by either the omission or intrusion of the child’s needs for care and support, has detrimental effects on an individual’s mental health in adulthood [44,61,84,110]. In particular, being exposed to a caregiver’s behavioral pattern appears to contribute to the development of deficits in reflective functioning [44,66,67,69]. This occurs because experiences of emotional abuse and neglect in childhood disrupt attachment strategies, preventing the child from using the caregiver as a secure base [44,74]. As a result, individuals may struggle to mentally represent and understand human behavior.

Moreover, as hypothesized, deficits in reflective functioning are positively associated with FA symptoms. Also, in this case, the results align with the literature. Indeed, individuals with problematic reflective functioning tend to face reality more instinctively and dysregulate, both emotionally and behaviorally [71,72,73,74]. In stressful and/or painful situations, individuals with reflective functioning deficits are more likely to use substances and/or disordered eating behaviors for self-regulation [74], thus rebalancing the individual’s emotional and cognitive state. This urge for an external object and/or substance (such as food) to achieve self-regulation is also a key behavior found in numerous studies related to overeating and binge eating [4,16].

In this regard, FA appears to act as a “natural bridge” between deficits in reflective functioning, substance use, and psychopathology, as it encompasses both the behavioral characteristics of an SRAD and an ED [1,41]. Indeed, FA primarily manifests, although not exclusively, through the consumption of food that is heavily characterized by the presence of HPFs [111]: ultra-processed products capable of eliciting and maintaining addictive behaviors, with biological, emotional, and psychological reactions similar to those seen with substance use. At the same time, FA also manifests through non-disordered eating behaviors such as grazing, binge eating, or uncontrolled eating, which are in turn associated with reduced dietary control [16].

Finally, in line with the literature, the results of the present study show a direct link between childhood emotional abuse and FA. Indeed, childhood emotional maltreatment has been linked to alterations in their neurocognitive systems [54,112]. In this regard, it is important to highlight the observed correlation between the two types of childhood emotional maltreatment and FA symptoms. In particular, the relationship between childhood emotional abuse and FA symptoms appears to be moderately strong. This association, in addition to the link between the variables already reported in the literature [42,53,54], could be due to both the ordinal nature of the data and the use of a convenience sample, which may have led to an overrepresentation of individuals with scores equal to 0—potentially inflating the strength of the association. Future studies should aim to confirm these findings. However, these results are consistent with literature [42,53,54]. Indeed, research suggests that those with a history of early-life trauma may exhibit reduced sensitivity to rewards, as evidenced by diminished subjective responses to reward-predicting cues and lower activation in striatal regions during reward anticipation [54,113]. This response may be interpreted as an adaptive mechanism.

In summary, the results of the present study show how experiences of emotional abuse in childhood have an indirect effect on both disordered eating behaviors related to excessive food consumption and reduced dietary control. These variables are linked through two bridging variables: deficits in reflective functioning and FA symptoms. In particular, the latter would trigger disordered eating behaviors, leading the individual to consume the substance they are dependent on (i.e., HPFs) to achieve emotional and psychological regulation.

### 4.1. Strengths and Limitations

This study presents some limitations. First, it relies on self-reported data without incorporating clinical interviews or behavioral assessments, which would allow for a more accurate evaluation of childhood emotional maltreatment or the severity of deficits in reflective functioning, as well as the extent of FA symptoms. Nevertheless, all the instruments used in this research are well-validated and reliably capture the intended constructs. Second, its cross-sectional nature prevents the establishment of causal relationships. However, this study is based on a strong theoretical framework that guided the structuring of the analysis model. In this regard, it is important to remark that some of these variables (such as childhood maltreatment) are immutable [42,59] and can only assume a limited number of roles—specifically, as predictors—within statistical models. However, future research should consider longitudinal designs to examine the long-term impact of these variables. Another limitation is the study’s focus on a restricted number of variables; additional factors such as impulsivity, emotion regulation difficulties, and insecure attachment might also contribute and should be further explored in future investigations. Additionally, the sample was slightly unbalanced in terms of gender, with a higher representation of females. This suggests that future studies should account for possible gender and ethnic differences. Another potential limitation is that this study considers only biological sex (male and female) and not gender. Future studies might consider evaluating similar models that assess the impact of gender, as well as other socio-demographic and physical variables. While the sample size was relatively large, findings are generalizable only to the Italian population and may be influenced by representation bias. Future research could aim to expand the sample to better reflect the overall Italian population or test these hypotheses in clinical populations, such as individuals with eating disorders or obesity. Moreover, it is worth emphasizing that the internal consistency values of both the emotional abuse and emotional neglect scales are not entirely satisfactory, which could suggest a low association between the items within the scale. However, it is important to highlight that internal consistency depends on the number of items [114]. Therefore, since each scale consists of only three items, it is unlikely that they would produce high internal consistency values, especially in the absence of semantic redundancy, as in this case. Additionally, these results are perfectly consistent with the literature [60], which reports similar internal consistency values. For these reasons, it is reasonable to consider the study’s findings as reliable and robust. However, future studies could benefit from using other instruments to investigate the presence of emotional maltreatment in childhood. Finally, since two of the scales used (YFAS 2.0 and AEBS) assess constructs related to excessive food consumption and overeating, there is a possibility of semantic overlap among some items, which could artificially inflate correlations between the scales. However, this is not the case. It is important to highlight that the correlations observed in this study remain below the critical threshold [96,97,98], and each scale assesses distinct aspects of eating behavior. Moreover, it is important to emphasize that the YFAS 2.0 assesses FA symptoms (such as tolerance, withdrawal, or craving), while the AEBS was designed to measure constructs related to food consumption that may result from the presence of FA [93], such as excessive food intake and reduced dietary control [33]. Therefore, concerns regarding redundancy are minimal, and the results can be considered reliable.

Despite these limitations, this research offers several strengths. First of all, as previously stated, this study is grounded in a robust theoretical framework that guided both the development of the analysis model and the rigorous methodological and statistical procedures. Also, this study included a large and diverse sample and employed reliable, validated measures. Additionally, to the best of our knowledge, this is the first study provides valuable insights into the relationships between emotional maltreatments and disorders eating behaviors highlighting the role of reflective functioning and FA symptoms. Moreover, it highlights the central role of psychological processes in overeating behavior and reduced diet control, aligning with key psychological principles underlying modern therapeutic approaches [72], such as mentalization-based treatment [115,116].

### 4.2. Clinical Implications

The findings of this study hold valuable implications for both research and clinical practice. From a research perspective, the study contributes to a deeper understanding of reflective functioning and FA by providing a solid empirical foundation for future investigations. On the clinical side, the implications align with the key points related to modern evidence-based psychotherapeutic approaches, such as MBT [117,118,119]. In particular, findings of the present study suggest that clinicians could focus on enhancing the ability to manage the behavioral and psychological consequences of deficits in reflective functioning [72,115,116,120,121,122]. By targeting dysfunctional thought patterns, emotional dysregulation, and maladaptive behaviors, these interventions aim to improve overall psychological well-being and could reduce disordered eating behaviors related to FA, perhaps in combination with specific structured protocols [123].

This study underscores the fundamental role of deficits in reflective functioning and FA in shaping the psychological trajectory from childhood emotional maltreatment to disordered overeating behaviors and reduced dietary control. By elucidating these connections, the findings offer valuable insights for clinicians working with individuals struggling with overeating, providing an initial framework for developing targeted interventions.

## 5. Conclusions

This study contributes to the understanding of disordered eating behaviors, including overeating and poor dietary control, associated with childhood emotional maltreatment through deficits in reflective functioning and FA. To the best of our knowledge, this is the first study to investigate this potential pathway, emphasizing the role of FA and its connection to deficits in reflective functioning. Furthermore, this study could pave the way for the implementation of new treatments for eating behaviors related to FA and its symptoms.

The findings indicate that deficits in reflective functioning, linked to emotional abuse and emotional neglect, are directly associated with FA and its related eating behaviors. These results highlight the significant role of childhood emotional trauma and impairments in reflective capacity in the development and persistence of dysfunctional eating behaviors associated with FA.

Unlike prior research, which mainly focused on proximal psychological or medical correlates of FA, this study offers a novel interpretation by integrating more distal developmental factors. By framing FA as a mediating mechanism linking early emotional maltreatment to dysregulated eating, the findings broaden the conceptualization of its etiology.

## Figures and Tables

**Table 1:** Sample descriptive statistics.

	Descriptives	
Age (mean *SD*)	38.39	14.70
BMI (mean *SD*)	24.38	4.86
Gender (*n %*)		
Male	116	21.4%
Female	427	78.6%
Civil status (*n %*)		
Single	132	24.3%
In a relationship	185	34.1%
Married	182	33.5%
Separated/divorced	36	6.6%
Widowed	8	1.5%
Education (*n %*)		
Middle school degree	29	5.4%
High school degree	237	43.6%
Bachelor degree	237	43.6%
Master/Ph.D.	40	7.4%
Work Status (*n %*)		
Student	147	27.1%
Full-time worker	210	38.7%
Part-time worker	64	11.8%
Entrepreneurs	65	12.0%
Unemployed	22	4.1%
Retired	35	6.4%
BMI Class (*n %*)		
Severely underweight (<16)	2	0.4%
Underweight (16–18.49)	19	3.5%
Normal weight (18.5–24.99)	331	61.0%
Overweight (25–29.99)	126	23.2%
Class I obesity (30–34.99)	43	7.9%
Class II obesity (35–39.99)	15	2.8%
Class III obesity (>40)	7	1.3%
ED diagnosis (*n %*)		
No ED	487	89.7%
Anorexia nervosa	12	2.2%
Bulimia nervosa	11	2.0%
Binge eating disorder	16	2.9%
ED no otherwise specified	17	3.1%

**Table 2:** Descriptive statistics, correlations among variables.

		Descriptives				Correlations						
		M	SD	SK	K	1	2	3	4	5	6	7
1	Emotional abuse	0.44	0.76	1.82	2.81	-						
2	Emotional neglect	0.24	0.56	2.80	8.87	0.408	-					
3	(deficits in) Reflective functioning	3.23	3.37	1.02	0.24	0.323	0.222	-				
4	YFAS2 Symptom Count	1.10	2.10	2.40	5.56	0.491	0.224	0.321	-			
5	Appetite drive	16.99	6.30	1.36	1.90	0.317	0.160	0.351	0.593	-		
6	Low diet control	16.22	4.79	0.10	−0.25	0.188	0.088 *	0.236	0.309	0.467	-	
7	BMI	24.38	4.86	1.48	3.39	0.227	0.141 *	0.061 §	0.266	0.361	0.222	-

*Note*: All correlations are statistically significant with *p* < 0.001, except for * *p* < 0.050 and § *p* > 0.050. M = mean, SD = standard deviation, SK = skewness, K = kurtosis.

**Table 3:** Summary of standardized parameter estimates (beta) with 95% confidence intervals of the tested model (Figure 2).

		β *	β (SE)	95%CI [L; U]	z-Value	*p*-Value	*R* ^2^
Emo. abuse (X1) → (deficits in) reflective func. (M1)	a11	0.284	1.256 (0.202)	[0.858; 1.660]	6.210	<0.001	0.119
Emo. neglect (X2) → (deficits in) reflective func. (M1)	a21	0.109	0.658 (0.275)	[0.109; 1.199]	2.395	0.017	
(deficits in) reflective func. (M1) → FA symptoms (M2)	d	0.187	0.114 (0.026)	[0.062; 0.165]	4.380	<0.001	0.275
FA symptoms (M2) → appetite drive (Y1)	b21	0.488	1.465 (0.154)	[1.155; 1.758]	9.514	<0.001	0.411
FA symptoms (M2) → low diet control (Y2)	b22	0.219	0.505 (0.115)	[0.287; 0.733]	4.381	<0.001	0.128
Appetite drive (Y1) ↔ low diet control (Y2)		0.324	6.837 (0.921)	[4.940; 8.558]	7.427	<0.001	
Emo. abuse (X1) ↔ Emo. neglect (X2)		0.408	0.175(0.042)	[0.097; 0.262]	4.138	<0.001	
Emo. abuse (X1) → FA symptoms (M2)	a12	0.404	1.092 (0.188)	[0.688; 1.438]	5.810	<0.001	
Emo. neglect (X2) → FA symptoms (M2)	a22	−0.000	−0.001 (0.223)	[−0.413; 0.452]	−0.005	0.996	
(deficits in) reflective func. (M1) → appetite drive (Y1)	b11	0.196	0.360 (0.075)	[0.212; 0.506]	4.802	<0.001	
(deficits in) reflective func. (M1) → low diet control (Y2)	b12	0.161	0.227 (0.067)	[0.095; 0.358]	3.369	0.001	
Emo. abuse (X1) → appetite drive (Y1)	c1	−0.027	−0.217 (0.392)	[−0.966; 0.561]	−0.554	0.580	
Emo. neglect (X2) → appetite drive (Y1)	c2	−0.012	−0.128 (0.457)	[−1.037; 0.763]	−0.279	0.780	
Emo. abuse (X1) → low diet control (Y2)	c3	−0.000	−0.002 (0.333)	[−0.653; 0.667]	−0.005	0.996	
Emo. neglect (X2) → low diet control (Y2)	c4	−0.019	−0.159 (0.419)	[−0.986; 0.652]	−0.380	0.704	
Sex → (deficits in) reflective func. (M1)		−0.018	−0.145 (0.350)	[−0.837; 0.533]	−0.413	0.680	
BMI → (deficits in) reflective func. (M1)		−0.021	−0.015 (0.026)	[−0.066; 0.037]	−0.572	0.567	
Sex → FA symptoms (M2)		−0.028	−0.142 (0.191)	[−0.518; 0.227]	−0.746	0.456	
BMI → FA symptoms (M2)		0.163	0.069 (0.020)	[0.029; 0.108]	3.412	0.001	
Sex → appetite drive (Y1)		−0.040	−0.611 (0.539)	[−1.674; 0.449]	−1.133	0.257	
BMI → appetite drive (Y1)		0.225	0.287 (0.051)	[0.195; 0.392]	5.659	<0.001	
Sex → low diet control (Y2)		0.005	0.061 (0.499)	[−0.925; 0.997]	0.122	0.903	
BMI → low diet control (Y2)		0.158	0.154 (0.043)	[0.067; 0.233]	3.630	<0.001	
Effect of X1 on Y1 via M1	a11*b11	0.056	0.452 (0.122)	[0.235; 0.708]	3.695	<0.001	
Effect of X1 on Y1 via M2	a12*b21	0.197	1.600 (0.307)	[0.998; 2.186]	5.207	<0.001	
Indirect effect of X1 on Y1 via M1 and M2	a11*d*b21	0.026	0.210 (0.062)	[0.102; 0.341]	3.386	0.001	
Total indirect effect of X1 on Y1		0.278	2.261 (0.341)	[1.593; 2.923]	6.636	<0.001	
Total effect of X1 on Y1		0.252	2.044 (0.444)	[1.158; 2.895]	4.604	<0.001	
Effect of X1 on Y2 via M1	a11*b12	0.046	0.285 (0.100)	[0.109; 0.493]	2.859	0.004	
Effect of X1 on Y2 via M2	a12*b22	0.088	0.551 (0.152)	[0.276; 0.863]	3.626	<0.001	
Indirect effect of X1 on Y2 via M1 and M2	a11*d*b22	0.012	0.072 (0.027)	[0.029; 0.133]	2.721	0.007	
Total indirect effect of X1 on Y2		0.146	0.908 (0.180)	[0.567; 1.289]	5.047	<0.001	
Total effect of X1 on Y2		0.145	0.906 (0.300)	[0.312; 1.496]	3.022	0.003	
Effect of X2 on Y1 via M1	a21*b11	0.021	0.237 (0.110)	[0.038; 0.470]	2.153	0.031	
Effect of X2 on Y1 via M2	a22*b21	−0.000	−0.002 (0.329)	[−0.605; 0.682]	−0.005	0.996	
Indirect effect of X2 on Y1 via M1 and M2	a21*d*b21	0.010	0.110 (0.055)	[0.015; 0.232]	1.981	0.048	
Total indirect effect of X2 on Y1		0.031	0.345 (0.372)	[−0.353; 1.104]	0.928	0.353	
Total effect of X2 on Y1		0.020	0.217 (0.549)	[−0.815; 1.310]	0.396	0.692	
Effect of X2 on Y2 via M1	a21*b12	0.018	0.149 (0.077)	[0.019; 0.321]	1.930	0.054	
Effect of X2 on Y2 via M2	a22*b22	−0.000	−0.001 (0.115)	[−0.216; 0.240]	−0.005	0.996	
Indirect effect of X2 on Y2 via M1 and M2	a21*d*b22	0.004	0.038 (0.021)	[0.005; 0.085]	1.843	0.065	
Total indirect effect of X2 on Y2		0.022	0.186 (0.148)	[−0.095; 0.494]	1.258	0.208	
Total effect of X2 on Y2		0.003	0.027 (0.420)	[−0.801; 0.850]	0.064	0.949	

*Note*: β * = standardized beta; β = unstandardized beta; SE = standard error; 95%CI = 95% confidence intervals (lower/upper) for the unstandardized beta; *R*^2^ = explained variance *R*^2^ = explained variance; Emo. Abuse = TEC emotional abuse scale; Emo. Neglect = TEC emotional neglect scale; (deficits in) reflective func. = RFQ-8 Uncertainty scale; FA symptoms = Symptom count of the YFAS 2; appetite drive = AEBS appetite drive scale; low diet control = AEBS low diet-control scale.

## Data Availability

Data are available on a reasonable request due to privacy and ethical restrictions.
